# The evolving landscape of rubella in the WHO African Region

**DOI:** 10.11604/pamj.supp.2025.51.1.47534

**Published:** 2025-06-17

**Authors:** Balcha Masresha, Richard Luce, Reggis Katsande, Goitom Weldegebriel, Charles Wiysonge

**Affiliations:** 1 World Health Organization, Regional Office for Africa, Brazzaville, Congo; 2 World Health Organization, Inter-country Team for Eastern and Southern Africa, Harare, Zimbabwe

**Keywords:** Rubella, epidemiology, elimination, WHO African Region

## Abstract

**Introduction:**

as of the end of 2024, a total of 35 countries in the World Health Organization (WHO) African Region have introduced rubella vaccine. Globally, rubella cases declined by 81% between 2012 and 2022. We looked at the overall trends of rubella occurrence, comparing the reported cases before and following rubella vaccine introduction.

**Methods:**

we analyzed the vaccination coverage as well as rubella surveillance and laboratory confirmation data from countries in the African Region.

**Results:**

the regional coverage with the first dose of rubella-containing vaccine was 36% in 2023. Between 2009 and 2024, a total of 27,954 rubella laboratory confirmed cases were reported through the case-based surveillance system from the 26 countries that had introduced rubella vaccine by 2018, of which 18% were reported in the years after the vaccine introduction. Among 19 of the 26 countries which had good surveillance performance, there was an average 76% reduction in the reported number of rubella cases following vaccine introduction.

**Conclusion:**

the level of reduction of rubella cases after vaccine introduction observed in this study is comparable to the findings from other regions. Maintaining high population immunity and good quality disease surveillance is critical to verify elimination.

## Introduction

Rubella is a mild and self-limiting viral infection. However, when rubella infection occurs during the first trimester of pregnancy, it can lead to abortions, intra-uterine fetal deaths, still births, or a constellation of clinical signs which start manifesting in the neonatal and infancy period as a result of the damage to developing fetal organs. These constitute what is known as congenital rubella syndrome (CRS) [1].

Countries in the World Health Organization (WHO) African Region have been implementing measles control strategies since 2001. Priority activities have included the implementation of measles-rubella surveillance with laboratory confirmation, and the introduction of rubella-containing vaccine (RCV) in their immunization schedules. The African Region of the WHO has established an objective to achieve the elimination of measles and rubella in at least 80% of the countries in the Region by 2030 [2].

Rubella surveillance was established beginning in 2002 for many counties and was linked to measles surveillance in that specimens testing IgM negative for measles undergo serological testing for rubella. As a result, this measles/rubella surveillance system with laboratory confirmation produces critical epidemiological data on rubella occurrence in many countries starting in the pre-vaccine period. Between 2002 and 2009 in the African region, a total of 25,631 confirmed rubella cases were reported, of which 76% were aged < 10 years and the mean age of the cases was 7.3 years [3]. A global level progress report indicated that reported rubella cases declined 81%, from 93,816 in 2012 to 17,407 in 2022. The African Region of the WHO reported 10021 (58%) of the 17407 cases in 2022 [4]. The same report states that, globally, verification of rubella elimination was achieved in 98 of 194 countries by 2022 [4]; however, no country in the African region has verified rubella elimination to date. Rubella circulation has been more persistent in countries that have delayed introduction of RCVs. For example, South Africa, which only introduced RCV in 2024, reported 6,643 laboratory-confirmed rubella cases between May 2015 and December 2019, with a median age of 5 years [5].

CRS sentinel surveillance is not widely implemented in the WHO African Region. Therefore, the actual burden of CRS is not well described. However, sporadic reports of CRS cases and rubella surveillance data indicate that rubella infection occurs among adults, making it probable that CRS is a public health concern even if not widely reported [5,6]. The lack of comprehensive CRS data has led to the use of disease modeling, which generates estimates showing that the African Region has the highest global CRS disease burden. Vynnycky et al estimated that for 2019, the African Region had a regional CRS incidence rate of 64 per 100,000 live births. The Eastern Mediterranean and South-East Asian Regions had estimated CRS incidence of 28 and <1 per 100,000 live births, respectively [7].

The WHO position paper for rubella vaccine was revised in 2024 to allow all countries to introduce rubella containing vaccine irrespective of their routine immunisation coverage with measles vaccine, removing prior requirements for countries to attain at least 80% MCV1 coverage according to the WHO UNICEF estimates or 80% coverage in Supplemental immunization Activities (SIAs) as confirmed by post-campaign survey [8]. The WHO recommended strategy for rubella vaccine introduction in countries with programmatic and coverage gaps includes doing an initial wide-age range catch-up vaccination campaign often targeting children 9 months to 15 years of age, followed by introduction into the routine immunisation programme. A single dose of rubella-containing vaccine can provide lifelong protection against rubella [9].

In the African region, GAVI, the Vaccine Alliance (GAVI) supported RCV introduction in 2013 in Ghana, Rwanda and Senegal followed by Burkina Faso and Tanzania in 2014. With the critical support of funding from GAVI, RCV introduction continued progressively until, as of December 2024, rubella vaccine was part of the routine immunisation schedule in 35 of the 47 countries in the African Region. Seychelles, Mauritius, Algeria and South Africa introduced rubella vaccine directly into the routine vaccination programme without an initial catch-up vaccination campaign, while the remaining countries have implemented the initial catch-up vaccination campaigns followed by introduction into the routine immunization system. The majority countries in the Region which have introduced rubella-containing vaccines use measles-rubella (MR) vaccine except Algeria, Cape Verde, Mauritius and Seychelles which use the measles-mumps-rubella (MMR) vaccine [10].

Various studies have documented the immediate decline in rubella incidence following RCV introduction. A global report indicated a decline in the total number of rubella cases from 93,816 in 2012 to 17,407 in 2022 [4]. In the WHO South-eastern Asia Region, rubella incidence declined by 80% over a period of 8 years following the introduction of RCV through SIAs in 10 countries and following overall Regional increase in RCV first dose (RCV1) coverage from 12% to 86% [11]. In the African Region, one study looked at the rubella epidemiology for 3 years following the catch-up vaccination campaigns in 5 countries and demonstrated a substantial post-campaign decline in the number of confirmed rubella cases and in the overall annual incidence of rubella [12].

WHO recommends that countries establish National Verification Committees to oversee the detailed documentation of national progress towards measles and rubella elimination. All countries that seek verification of the elimination of measles or rubella are expected to provide compelling data that circulation of the endemic virus has been interrupted for at least 36 months, in the presence of a high-quality surveillance system [13]. The WHO African Region has established a Regional Commission for the verification of measles and rubella elimination. Since 2018, 13 countries have established national committees and started to document their progress as per the standard lines of evidence. In this study, we analyze the overall trends of rubella occurrence in the WHO African Region between 2010 and 2024, as well as the trends in 26 RCV-using countries spanning at least five years following rubella vaccine introduction, including five countries that have 10 years of data following the introductory campaign.

## Methods

We used the WHO and UNICEF estimates of national vaccination coverage (WUENIC) for all vaccine doses which are generated using annual administrative coverage data, national official coverage estimates, and vaccination coverage surveys. We reviewed the WUENIC coverage for RCV1 for the region [14].

We analyzed case-based surveillance data for the period from 2010 to 2024 for the African Region, and specifically the data for 26 countries that introduced RCV vaccine between 2013 and 2018. Rubella surveillance data is collected as part of the measles-rubella case-based surveillance system which has been operational in the African Region since 2004. The case definition used in many countries for reporting suspected cases in the surveillance system is the case definition for suspected measles cases, while a few countries near to measles elimination use the generalized febrile rash illness case definition [15]. Each suspected case undergoes case-based investigation to collect relevant epidemiological information and blood specimens to test for measles-specific and rubella-specific immunoglobulin M (IgM) antibody using a standard enzyme-linked immunosorbent assay. Many national measles laboratories conduct parallel testing while some only test the measles IgM negative specimens for the presence of rubella-specific IgM antibody. Specimens are processed in national or subnational measles/rubella serological laboratories, which, as part of the Regional and Global network, utilize standardized methods, and undergo regular quality control and accreditation by WHO [16].

At the WHO African Regional level, national measles-rubella case-based surveillance performance is monitored regularly using two indicators - the non-measles febrile rash illness rate (NMFRI) and the proportion of districts reporting [15]. For the calculation of the average number of annual rubella case reports, the pre-introduction period was the years before the introduction of rubella vaccine and the year of the initial MR catch-up vaccination campaigns - also referred to as supplemental immunisation activities (SIAs). The post-introduction period was the years following the initial MR catch-up campaigns. The mean annual number of rubella cases was calculated by taking the number of rubella cases reported during the specific period of years in consideration divided by the number of years.

Our study included a review of the epidemiological data across all 47 countries in the Region, and a specific detailed focus on the trends before and after vaccine introduction in 26 countries that had introduced RCV by 2018, excluding countries with very small number of rubella case reports (Comoros, Cabo Verde, and Sao Tome and Principe), and countries which had introduced RCV directly into the routine immunisation programme before 2013 without having done catch-up SIAs (Algeria, Mauritius and Seychelles).

The 26 countries in our series implemented RCV catch-up vaccination campaigns at different times. We analyzed the average annual number of rubella cases reported in the 5 years preceding to and including the year of the catch-up campaign, comparing it to the average rubella cases reported following the campaign, until 2024. The 5-year pre-campaign period was selected since this is the minimum number of pre-SIAs data we have from all 26 countries during this period 2009 - 2024. The post-campaign period ranged from 5 years to 11 years depending on the year of the catch-up SIAs.

## Results

From the latest available WHO UNICEF estimates of vaccination coverage for 2023, RCV1 coverage is 36% at African Regional level, up from 3% in 2013 - the year of the first MR catchup SIAs (Table 1). As of the end of 2023 the following 15 countries had not introduced RCV in their immunization schedules: Central African Republic, Chad, Democratic Republic of Congo (DR Congo), Equatorial Guinea, Ethiopia, Gabon, Guinea, Guinea Bissau, Liberia, Mali, Madagascar, Niger, Nigeria, South Africa, South Sudan. By 2024, Mali implemented an MR catch-up campaign and introduced MR in the routine immunization program, while South Africa introduced MR only in the childhood routine immunization schedule.

**Table 1:** measles and rubella vaccination coverage in the WHO African Region (WHO-UNICEF coverage estimates 2010-2023)

	First dose measles containing vaccine coverage (MCV1)	Second dose measles containing vaccine coverage (MCV2)	First dose rubella containing vaccine coverage (RCV1)
**2010**	72%	4%	0%
**2011**	70%	4%	0%
**2012**	70%	6%	0%
**2013**	69%	7%	3%
**2014**	69%	10%	9%
**2015**	68%	17%	11%
**2016**	68%	22%	12%
**2017**	69%	24%	25%
**2018**	70%	25%	31%
**2019**	71%	33%	32%
**2020**	69%	39%	35%
**2021**	67%	40%	34%
**2022**	68%	44%	35%
**2023**	70%	49%	36%

**Figure 1:**
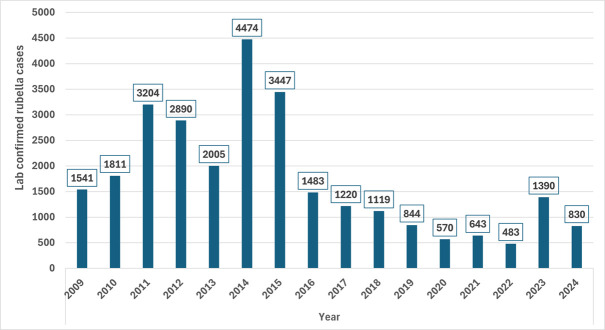
laboratory confirmed rubella cases reported from 2009-2024 from 26 countries that introduced RCV by 2018 (African Region)

In 2023, there were a total of 4694 rubella confirmed cases reported from the 47 countries in the Region, of which 1378 (71%) were reported from the 15 (32%) countries who had not yet introduced the vaccine at the time. In 2024, South Africa reported 11,705 confirmed rubella cases comprising of 82% of all the reported 14340 rubella cases in the Region for the year. Excluding this large outbreak from South Africa, analysis of the case reports from the remaining countries indicates that 1745 (66%) of the 2635 cases were reported from the 13 countries who had not yet introduced rubella vaccine by 2024, with a mean incidence of 4.3 per million population. These 13 countries comprise 49% of the regional population. The 33 countries with RCV (excluding South Africa) had a mean rubella incidence of 1.4 per million population and a median of 0.9 per million population in 2024. Between 2009 and 2024, a total of 27,951 rubella laboratory confirmed cases were reported through the case-based surveillance system from the 26 countries that had introduced RCV between 2013 and 2018 in the Region. Out of this total, only 4933 cases (17.6%) were reported in the post-campaign years. The highest number of cases were reported in 2014, and the lowest was reported in 2022 (Figure 1).

Uganda and Kenya reported the highest number of cumulative number of cases - a total of 4430 and 3571 cases respectively during this period. The reported number of rubella cases indicated an increase in 2023 in Tanzania (440 cases), Zimbabwe (136 cases), Cote d’Ivoire (114 cases), Angola (85 cases), Uganda (77 cases) and Botswana (62 cases), as compared to the preceding years (Table 2). In comparing the average annual number of case reports pre- and post-campaign, we excluded 7 countries (Angola, Benin, Burundi, Malawi, Mauritania, Sierra Leone and Zambia) because of gaps in their surveillance performance; the countries missed the target for the two principal surveillance performance indicators for 5 or more years in the 16 years period. The analysis on the remaining 19 countries shows that there was an average 76% reduction in the number of rubella cases (median 87%). The range in reduction of rubella cases varies from 8% to 97%. Twelve of the 19 countries had more than 80% reduction in the average annual number of rubella cases reported post-SIAs (Table 3). For the 5 countries that have data for at least 10 years following the catch-up campaign, the reduction in case load has been sustained, with Ghana and Burkina Faso having >80% reduction. Rwanda and Senegal have attained 76% and 51% reduction respectively, while Tanzania has only 8% reduction.

**Table 2:** laboratory confirmed rubella cases by country 2009-2024 (the year of RCV catch-up campaigns are shaded)

Country	Year															
	2009	2010	2011	2012	2013	2014	2015	2016	2017	2018	2019	2020	2021	2022	2023	2024
**Angola**	2	0	5	65	31	112	288	12	20	32	69	11	35	38	85	0
**Benin**	15	1	11	41	10	8	32	3	0	10	1	9	6	22	31	2
**Botswana**	4	13	193	24	104	441	5	11	3	3	4	7	0	1	62	4
**Burkina Faso**	12	84	52	98	27	57	4	1	1	4	1	8	1	0	10	3
**Burundi**	42	29	17	11	7	70	61	18	2	6	4	14	17	13	37	40
**Cameroon**	32	44	103	147	147	147	279	15	12	7	49	21	36	25	10	17
**Congo**	10	10	25	22	214	45	4	2	12	122	37	3	15	1	5	4
**Cote d'Ivoire**	50	31	38	232	87	54	50	192	329	197	77	122	80	27	114	86
**Eritrea**	0	47	0	17	15	32	14	44	2	5	11	19	8	12	5	2
**Eswatini**	89	94	0	23	0	1	2	4	0	0	4	2	0	0	1	3
**Gambia**	0	10	40	36	59	30	1	2	0	1	0	3	1	6	13	5
**Ghana**	114	160	486	367	168	39	12	10	7	20	14	36	42	59	92	0
**Kenya**	415	472	597	300	299	581	423	257	14	24	62	21	14	28	50	14
**Lesotho**	72	78	0	68	166	294	129	11	8	0	4	0	0	9	9	2
**Malawi**	40	13	21	44	15	385	81	28	25	6	6	0	0	0	5	59
**Mauritania**	33	2	20	0	3	48	9	0	0	1	0	0	0	0	4	3
**Mozambique**	63	69	124	428	108	207	358	83	48	110	72	138	243	48	83	60
**Namibia**	14	157	120	42	12	84	415	37	17	5	8	4	1	5	10	5
**Rwanda**	36	36	31	172	50	11	1	15	22	11	0	31	29	2	12	36
**Senegal**	55	11	141	44	26	101	15	19	7	8	22	5	34	28	30	30
**Siera Leone**	52	2	9	21	4	0	11	97	355	6	0	0	3	1	5	1
**Tanzania**	29	124	18	30	107	300	29	23	28	24	39	49	17	71	440	340
**Togo**	85	80	68	33	38	17	57	274	37	38	2	2	5	3	14	24
**Uganda**	177	132	581	472	159	292	1055	289	251	470	342	31	40	21	77	41
**Zambia**	75	14	56	136	61	90	92	34	16	8	0	20	15	21	50	30
**Zimbabwe**	25	98	448	17	88	1028	20	2	4	1	16	14	1	42	136	16

**Table 3:** change in the average annual rubella case reports by country before and after the vaccine introduction (African Region)

Country	Average annual number of rubella cases reported			Year of catch-up SIAs	# of years post SIAs including 2024
	5 years pre SIAs average	average post SIAs	% reduction		
**Botswana**	117	11	91%	2016	8
**Burkina Faso**	64	3	95%	2014	10
**Cameroon**	165	21	87%	2015	9
**Congo**	35	6	84%	2019	5
**Cote d'Ivoire**	164	84	49%	2018	6
**Eritrea**	19	10	47%	2018	6
**Eswatini**	6	1	83%	2016	8
**Gambia**	26	4	85%	2016	8
**Ghana**	259	30	88%	2013	11
**Kenya**	372	28	92%	2016	8
**Lesotho**	122	3	97%	2017	7
**Mozambique**	161	107	33%	2018	6
**Namibia**	118	7	94%	2016	8
**Rwanda**	65	15	77%	2014	11
**Senegal**	55	27	51%	2014	11
**Tanzania**	116	106	8%	2015	10
**Togo**	85	8	90%	2018	6
**Uganda**	481	42	91%	2019	5
**Zimbabwe**	320	26	92%	2015	9

## Discussion

The overall trends in rubella case reporting in these countries show a sustained significant decline since 2016, with the lowest case reports in 2020-2022. The apparent spike in rubella cases in 2023 and 2024 indicates that the reporting in 2020-2023 may have been affected by the COVID pandemic. There is also a possibility of declines in routine immunisation coverage or delays in periodic SIAs during the pandemic contributing to this increase in rubella cases in 2023-2024. From among the 5 countries with 10 years data following RCV introduction, we note that Tanzania had only 8% reduction in rubella cases in the post-campaign period. Tanzania reported rubella outbreaks in 2023 and 2024 with a total of 440 and 340 confirmed cases per year respectively, while the preceding post-campaign years had an average of 35 cases per year [17]. The negative impact of the COVID-19 pandemic on routine immunization, and the prolonged interval between Tanzania’s 2019 and 2024 follow-up vaccination campaigns may have contributed to the accumulation of susceptible young children that led to the increase in rubella cases.

Many studies have documented declines in disease surveillance quality and disruptions in routine immunization systems during the COVID pandemic period, which may have resulted in more unvaccinated children leading to increased virus circulation in subsequent years [18-20]. In addition to the catch-up SIAs and routine introduction of MR vaccine, countries implement periodic MR follow-up SIAs targeting the youngest birth cohorts in order to close the immunity gaps in children that have not received their routine doses. These periodic SIAs are implemented based on the calculation of the rate of accumulation of measles susceptible populations among under five age groups, and is often implemented every 2-5 years, depending on the routine immunisation and SIAs coverage levels of preceding years. In addition to the coverage gaps in routine immunization, any delays or gaps in the implementation of periodic SIAs risk exposing unvaccinated infants and young children to measles and rubella infection.

The level of post-campaign reduction of rubella cases observed in this study is comparable to the findings from the WHO South-East Asia and Western Pacific Regions [11,21]. Similarly, Namibia documented a rapid reduction in rubella and measles incidence following a wide age range campaign implemented in 2016. The average annual rubella incidence decreased by 95% in the years 2017-2023 as compared to the annual average in 2010-2016 [22]. Due to the high seroconversion for rubella vaccination following one dose of vaccine, and the lower infectivity of rubella as compared to measles, it has been documented that the elimination of rubella and CRS can be achieved much faster than that of measles. Indeed, the American Region of the WHO achieved rubella/CRS elimination before measles elimination [23]. As of the end of 2022, a total of 98 countries had attained rubella and CRS elimination globally [4]. Countries in the African region can benefit from the introduction of RCV to enhance their measles elimination activities, including through the initial wide age range MR vaccination campaigns which also help to address the measles immunity gaps among older children.

Maintaining high-quality disease surveillance is critical in all countries. In addition, high-performing countries are encouraged to implement elimination standard measles-rubella surveillance, which employs a broader fever and rash case definition to improve sensitivity, as well as the requirement for intensive investigation of all cases and clusters of febrile rash illnesses. Molecular surveillance is an important tool for determining prevalent circulating genotypes within countries. More importantly, it plays a key role in tracking importations, monitoring the elimination process and verifying the interruption of measles as well as rubella virus transmission [13]. The documentation of rubella genotypes remains very limited in countries in the African Region. National surveillance programmes and serological laboratories should strive to develop their molecular surveillance capacities and establish the baseline data to conclusively demonstrate and distinguish ongoing circulation from imported cases, which is critical in the lead up to national verification of measles and rubella elimination.

## Conclusion

Countries in the African Region have attained significant and sustained reduction of rubella cases after vaccine introduction. Maintaining high population immunity and good quality disease surveillance will be critical to verify elimination. This study has some limitations. Rubella is a mild illness and is often underreported. In addition, rubella reporting relies on the measles surveillance system and many countries still use the measles reporting definition for the initial notification and investigation of cases. This may exclude some rubella cases from being captured by the disease surveillance system. The sensitivity of disease surveillance determines the completeness of reporting of suspected cases. Even though we excluded from our analysis countries with obvious surveillance gaps, there may be other gaps in reporting which may limit the ability to fully capture the rubella epidemiological situation in some countries.

### What is known about this topic

- Rubella infection in early pregnancy can cause fetal organ damage and manifest as congenital rubella syndrome;
- An increasing number of countries in the African Region have introduced rubella vaccine in their national immunization schedules since 2013;
- No country in the African region has attained the verification of rubella elimination to date.

### What this study adds

- The first dose of rubella vaccination coverage in the African Region has increased from 3% in 2013 to 36% in 2023;
- One third of the countries in the region, comprising of those that have not yet introduced rubella vaccine, continue to report more than two thirds of rubella cases in 2023 and 2024;
- Countries in the African Region have documented sustained reduction in rubella cases following the vaccine introduction.
